# Crosstalk between stressed brain cells: direct and indirect effects of ischemia and aglycemia on microglia

**DOI:** 10.1186/s12974-020-1697-8

**Published:** 2020-01-24

**Authors:** Monika Rabenstein, Sabine Ulrike Vay, Stefan Blaschke, Helene Luise Walter, Anne Ladwig, Gereon Rudolf Fink, Maria Adele Rueger, Michael Schroeter

**Affiliations:** 1 Department of Neurology, Faculty of Medicine and University Hospital, University of Cologne, Kerpener Strasse 62, 50924 Cologne, Germany; 2 Research Centre Juelich, Cognitive Neuroscience, Institute of Neuroscience and Medicine (INM-3), Juelich, Germany

**Keywords:** OGD, Oxygen-glucose deprivation, Neurons, Astrocytes, preconditioning, inflammation, LPS, neural stem cells

## Abstract

**Background:**

In cerebral ischemia, microglia have a dichotomous role in keeping the balance between pro- and anti-inflammatory mediators to avoid deleterious chronic inflammation and to leverage repair processes.

**Methods:**

We examined functional and inflammatory markers in primary rat microglia in vitro after oxygen-glucose deprivation (OGD) or glucose deprivation (aglycemia). We then investigated the preconditioning effect of OGD or aglycemia upon a subsequent strong inflammatory stimulus, here lipopolysaccharides (LPS). Moreover, an “in vitro brain model” of neurons and glia, differentiated from primary rat neural stem cells, was exposed to OGD or aglycemia. Conditioned medium (CM) of this neuronal/glial co-culture was then used to condition microglia, followed by LPS as a “second hit.”

**Results:**

OGD or aglycemia at sublethal doses did not significantly affect microglia function, including the expression of inflammatory markers. However, preconditioning with either OGD or aglycemia led to a decreased pro-inflammatory response to a subsequent stimulus with LPS. Interestingly, the anti-inflammatory markers IGF-1 and IL-10 were additionally reduced after such preconditioning, while expression of CD206 remained unaffected. Treatment with CM from the neuronal/glial co-culture alone did not affect the expression of inflammatory markers in microglia. In contrast, treatment with CM increased the expression of both pro- and anti-inflammatory markers in microglia upon a second hit with LPS. Interestingly, this effect could be attenuated in microglia treated with CM from neuronal/glia co-cultures preconditioned with OGD or aglycemia.

**Conclusions:**

Data suggest specific and distinct microglia signatures in response to metabolic stress. While metabolic stress directly and indirectly applied to microglia did not mitigate their subsequent response to inflammation, preconditioning with metabolic stress factors such as OGD and aglycemia elicited a decreased inflammatory response to a subsequent inflammation stimulus.

## Background

Microglia, the innate immune cells of the central nervous system, demonstrate a highly dynamic, context-dependent phenotype expression determined by the local environment and recent exposure to stimuli [1, 2]. While in the past, microglia phenotypes were classified dichotomously as either the pro-inflammatory M1 or the anti-inflammatory M2 type, recent data reveal that microglia phenotypes are more complex and instead comprise a range of activation states [3]. We previously observed that microglia can switch between phenotypes upon external stimuli, while keeping a “memory” of the previous state [4]. Increasing evidence suggests that microglia may even adapt disease-specific phenotypes, e.g., in Alzheimer’s disease, amyotrophic lateral sclerosis, Parkinson’s disease, multiple sclerosis, and traumatic brain injury [5–7]. After a stroke, microglia develop a specific phenotype that can be distinguished from macrophages [8–10]. In post-ischemic inflammation, microglia have both beneficial and detrimental effects. On the one hand, they support regeneration by being neuroprotective and inducing repair processes, and on the other hand, they can potentiate secondary tissue damage if their pro-inflammatory activation does not cease [2, 11]. Further, microglia closely interact with astrocytes and neurons [6, 12]. Under the hypothesis that this crosstalk between microglia and other cells of the brain is crucial to microglia activation and polarization, and that microglia “memorize” this crosstalk, resulting in divergent responses to subsequent stimuli, we investigated primary rat microglia, as well as co-cultures from neurons and astrocytes, under oxygen-glucose deprivation (OGD) as an in vitro model of cerebral ischemia.

## Material and methods

All animal procedures followed the German Laws for Animal Protection and had been approved by the local animal care committee (Tierschutz-Beauftragte University of Cologne) and local governmental authorities (LANUV NRW, AZ 4.16.021).

### Cell culture

Microglia cultures were prepared from the cortices of rat pups 2 days postpartum as described previously [12]. Briefly, cells were trypsinized and distributed in flasks and allowed to grow there for 8–10 days in Dulbecco’s modified Eagle’s medium (DMEM) with the addition of 10% fetal calf serum (FCS), 1% penicillin/streptomycin, and 2 mM L-glutamine. After 8–10 days, cultures were shaken for 1 h at 250 rpm to detach microglia. The medium containing the layer of detached microglia was collected and centrifuged. The obtained microglia pellet was re-suspended in fresh culture medium and seeded into subcultures. Experiments were started at 24 h after cultivation.

Primary neural stem cells (NSC) were cultured from fetal rat cortices at embryonic day 13.5 as serum-free monolayers [13]. Briefly, cells were plated on dishes coated with poly-L-ornithine and fibronectin and expanded in Dulbeccos’s modified Eagle’s/F12 medium plus N2 supplement, penicillin/streptomycin, L-glutamine, and sodium pyruvate. As a mitogen, fibroblast growth factor (FGF)2 was included at 10 ng/ml. After first passaging, homogenous NSC cultures were re-plated in the presence of FGF2 at 10,000 cells per cm^2^. Only NSC from the second until the fourth passage were used for all experiments, in order to utilize unaltered primary cells.

The differentiation of NSC was induced after the withdrawal of FGF2 during the expansion phase. After 7 days, the culture medium was replaced by Neurabasal Medium with GlutaMAX, penicillin/streptomycin, L-glutamine, sodium selenite, B27, NT3, and BDNF. After 14 days of mitogen withdrawal, NSC had differentiated to a neuronal/glial co-culture and were used for experiments.

### Oxygen-glucose deprivation/aglycemia

We exposed microglia or the neuronal/glial co-culture to either combined oxygen-glucose deprivation (OGD) or glucose-deprivation alone (aglycemia). Microglia were exposed to this metabolic stress 1 day after subculturing, and neuronal/glial co-cultures were treated after 14 days of mitogen withdrawal from the initial NSC culture. To induce OGD, the culture medium was removed and cells were gently rinsed with PBS, then the medium was replaced by DMEM without glucose, and cells were placed in a hypoxic chamber (oxygen below 1 mmHg; Electrotek, Shipley, UK) at 37 °C (OGD) or in the incubator at normoxia (aglycemia). Oxygen concentration was measured with an oxygen meter (GMH 3611-GL, Greisinger, Regenstauf, Germany), the oxygen concentration was below 0.5% throughout all the experiments. After 30 min (sublethal OGD or aglycemia), the (deoxygenated) aglycemic solution was replaced by a fresh medium, and cells were allowed to recover under normoxia (5% CO2) at 37 °C for 20 h. Under control conditions, cells remained unstressed (i.e., untreated). The supernatant of the neuronal/glial co-culture was collected 20 h after OGD and stored at − 20 °C for further use. Microglia subjected to OGD or aglycemia underwent additional experiments (see below).

### Conditioned medium

To investigate the effects crosstalk between the neuronal/glial co-culture and microglia, the frozen supernatant of neuronal/glial co-cultures that underwent OGD or aglycemia was shortly thawed in a water bath at 37 °C and then incubated on subcultured microglia for 20 h.

### LPS treatment

To expose microglia to a strong inflammatory stimulus, cells were stimulated with 10 ng/ml LPS (derived from *E. coli* 0111: B4, cat. L4391, Sigma-Aldrich, St. Louis, MO, USA), either 20 h after OGD or aglycemia for 4 h (Fig. 2a) or after 20 h of incubation with conditioned medium of neuronal/glial co-cultures that underwent OGD or aglycemia (Fig. 4a). Afterwards, the supernatant was removed from the microglia and kept at − 20 °C, while cells were either fixed with 4% paraformaldehyde (PFA), or RNA was extracted.

### Immunocytochemistry

Representative images were taken using a digital microscope (Keyence BZ-9000E, Osaka, Japan).

#### Characterization of microglia

Characteristics of microglia in culture were confirmed immunocytochemically to verify the homogeneity of the cultures. Cells were fixed with 4% paraformaldehyde (PFA) and stained for ionized calcium-binding adapter molecule 1 (Iba1; rabbit polyclonal, dilution 1:1000, cat. 019- 19741, WAKO, Osaka, Japan). Microglia were stained for iNOS (mouse monoclonal, dilution 1:1000, cat. 49999, Abcam, Milton, UK) to detect effects of OGD or LPS treatment on the expression of iNOS. For visualization, fluorescein-labeled anti-mouse IgG or anti-rabbit IgG were used (dilution 1:200, Invitrogen, Karlsruhe, Germany); all cells were additionally counterstained with Hoechst 33342 (Life Technologies, Darmstadt, Germany).

#### Characterization of neuronal/glial co-cultures

The differentiation fates of NSC 14 days after mitogen withdrawal and the effects of OGD on those cultures were characterized immunocytochemically. Cells were fixed in 4% PFA and then stained for markers for neurons (neuron-specific beta-III tubulin: anti-TuJ1; mouse monoclonal, dilution 1:100, R&D Systems) and astrocytes (glial fibrillary acidic protein: anti-GFAP; rabbit polyclonal, dilution 1:2500, Abcam). For visualization, fluorescein-labeled anti-mouse IgG or anti-rabbit IgG were used; all cells were additionally counterstained with Hoechst 33342.

### Propidium iodide/Hoechst staining

Dead cells were stained with propidium iodide (Life Technologies, Darmstadt, Germany) to assess the effects of OGD, aglycemia, or treatment with conditioned medium of neuronal/glial co-cultures on the viability of microglia. All cells, irrespective of viability, were counterstained with Hoechst 33342 (Life Technologies, Darmstadt, Germany), and representative pictures were taken using an inverted fluorescence phase-contrast microscope (Keyence BZ-9000E). Five images were taken per well of a 24-well plate, and both Hoechst-stained and propidium iodide-stained microglia were counted manually. The number of propidium iodide-negative cells was divided by the total (Hoechst-stained) number of microglia in each sample to calculate the ratio of viable cells. The mean values were established among equally treated samples. Results were expressed as percent of the control ± SEM. *N* = 8–12 cell culture wells per condition from at least two independent experiments (cell culture preparations) were included in the analyses.

### Real-time quantitative PCR (RT-qPCR)

To quantify the effects of OGD, aglycemia, or treatment with conditioned medium of neuronal/glial co-cultures, on the inflammatory profile of microglia, RNA was collected at the end of the experiments and RT-qPCR was performed. RNA was isolated from cells by using the RNeasy Mini Kit (Qiagen, Hilden, Germany) following the manufacturer’s protocol. Total RNA concentration and purity were evaluated photometrically. Total RNA was converted to cDNA by reverse transcription the GeneUP using the total RNA mini Kit (Biotechrabbit, Henningsdorf, Germany). All primers were obtained from Biolegio (Nijmegen, The Netherlands). The sequences of the primers are listed in Table 1. The RT-qPCR reaction was carried out using 10 ng total RNA in a 20-μl reaction Quantitect Reagents (Qiagen, Hilden, Germany) following the manufacturer’s recommendations. The samples were amplified and quantified on a Bio-Rad cycler 006537 (Bio-Rad, Hercules, CA, USA). PCR product integrity was evaluated by melting point analysis and agarose gel electrophoresis. The threshold cycle (CT) was normalized to ribosomal protein L13a (RPL13a; ΔCT) and the experimental control condition (ΔΔCT). Mean fold changes are depicted as 2^(−ΔΔCT)^. Mean values ± SEM were calculated for all samples. *N* = 12 cell culture wells per condition from at least three independent experiments (cell culture preparations) were included in the analyses.

**Table 1:** Primers and parameters of RT-qPCR

RNA	Sequences forward/backward 5′-3′	Temperature (°C), step 1/2/3	Duration (s), step 1/2/3
iNOS	GCTTGTCTCTGGGTCCTCTG/ CTCACTGGGACAGCACAGA	95/59/72	15/15/45
CD206	AACAAGAATGGTGGGCAGTC/ CCTTTCAGTCCTTTGCAAGC	95/56/72	15/15/45
TNFα	CATCCGTTCTCTACCCAGCC/ AATTCTGAGCCCGGAGTTGG	95/56.6/72	15/15/45
IL-6	CCCAACTTCCAATGCTCTCCT/ AGCACACTAGGTTTGCCGAG	95/57.3/72	15/15/45
IL1-β	GACTTCACCATGGAACCCGT/ GGAGACTGCCCATTCTCGAC	95/56/72	15/15/45
IL-10	GAAAAATTGAACCACCCGGCA/ TTTCCAAGGAGTTGCTCCCG	95/56/72	15/15/45
RPL13a	TCTCCGAAAGCGGATGAACAC/ CAACACCTTGAGGCGTTCCA		15/15/45

### Phagocytosis assay

A phagocytosis assay was performed (CytoSelectTM 96-well Phagocytosis Assay, Cell Biolabs, San Diego, CA, USA) according to the manufacturer’s protocol to assess the effects of OGD or aglycemia on the phagocytic activity of microglia. Phagocytic activity was measured by the amount of engulfed prelabeled zymosan substrate uptake after 2 h of incubation, which was colorimetrically detected after blocking external zymosan particles. The optical density (OD) of each sample was measured at 405 nm in a plate reader (FLUOstar Omega, BMG LABTECH, Ortenberg, Germany). The resulting mean values ± SEM were established among equally treated samples and compared to microglia not exposed to zymosan. *N* = 6 cell culture wells per condition from at least two independent experiments (cell culture preparations) were included in the analyses.

### IGF1-ELISA

Insulin-like growth factor 1 (IGF1) was measured in the supernatant of microglia using the rat IGF1 Quantikine ELISA Kit (Cat#MG100, R&D systems, Minneapolis, Canada). Experiments were conducted according to the manufacturer’s protocol. The OD of each sample was measured at 450 and 570 nm in a plate reader (FLUOstar Omega, BMG LABTECH, Ortenberg, Germany), and cytokine concentrations of the samples were calculated based on standard curves. Mean values ±SEM were established among equally treated samples. *N* = 12 cell culture wells per condition from at least two independent experiments (cell culture preparations) were included in the analyses.

### Statistical analyses

Descriptive statistics were performed with Graph Pad Prism (GraphPad Software Inc.). For comparison of multiple groups, one-way analysis of variance (ANOVA) and Dunnet’s or Tukey’s post hoc tests were performed with the same software. Statistical significance was set at the < 5% level (*p* < 0.05).

## Results

### Microglia after OGD or aglycemia

**Fig. 1:**
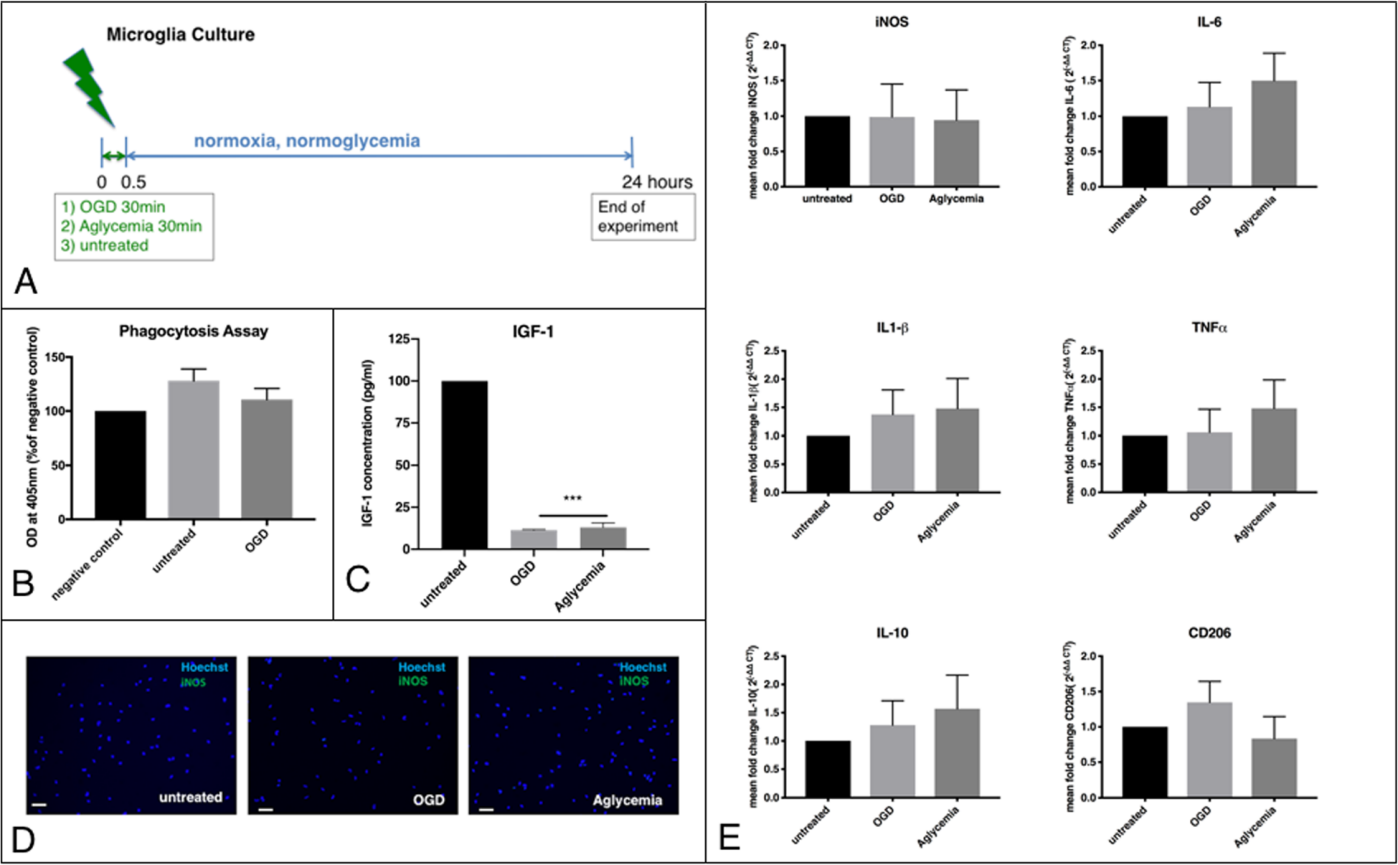
Microglia dynamics after metabolic stress. **a** Experimental timeline. Microglia were exposed to metabolic stress by OGD or aglycemia for 30 min and then allowed to recover in regular culture medium for 24 h. Then the cells were used for further experiments. Unstressed microglia served as controls. **b** Microglia were incubated with phagocytic beads (zymosan) for 2 h, and zymosan uptake was quantified photometrically. Untreated cells without zymosan incubation served as the negative control. There was no significant effect of OGD on the phagocytic activity of microglia compared to unstressed control cells (values displayed as means ± SEM of three independent experiments with *n* = 6/each condition). **c** An IGF1-ELISA was used to quantify IGF1 release in the cell supernatant photometrically. Both OGD and aglycemia significantly reduced IGF1 release compared to unstressed controls (values are displayed as means ± SEM of three experiments with *n* = 12/each condition; the difference between treated groups: ***p < 0.0001 (one-way ANOVA/Tukey’s multiple comparison test). **d** Representative immunocytochemical images of iNOS+ cells (green), co-stained with a nuclear marker (Hoechst; blue). Staining did not reveal any iNOS+ cells in either unstressed microglia (left panel), microglia after OGD (middle panel), or microglia after aglycemia (right panel) (scale bars = 50 μm). **e** Q-PCR revealed that neither OGD nor aglycemia affected the expression of the M1 and M2 markers iNOS, TNFα, IL-6, IL-1β, IL-10, and CD 206 compared to unstressed microglia (values displayed as means ± SEM of three independent experiments with *n* = 12/ each condition, one-way ANOVA). Each Q-PCR sample was normalized to RPL13a as the reference gene, and mRNA levels were normalized to endogenous RPL13a expression

The cells were exposed to 30 min of either OGD or aglycemia alone to investigate the direct effects of metabolic stress on microglia. Afterwards, they were allowed to recover for 20 h at normoglycemic and normoxic conditions (Fig. 1a). Metabolic stress for 30 min did not affect microglia viability, while OGD at a longer duration of 45 min led to significantly increased cell death (Additional file 1: Figure S1A). Microglia function after metabolic stress was assessed by phagocytosis assay, revealing no significant change in phagocytic activity between the stressed groups and unstressed controls (Fig. 1a). Remarkably, microglia exposed to OGD or aglycemia significantly reduced the production of IGF-1 as an anti-inflammatory cytokine, compared to unstressed controls (Fig. 1c). However, iNOS production as a pro-inflammatory marker was not affected by OGD or aglycemia, neither on the protein level, as detected by immunocytochemistry (zero to one iNOS+ cells were found in the three conditions, due to the low amount no further quantification was carried out) (Fig. 1d), nor on the RNA level, as detected by qPCR (Fig. 1e). Further quantification of pro- and anti-inflammatory markers by qPCR revealed no changes in the mRNA expression of TNFα, IL-6, IL1-β, IL-10, and CD 206 upon OGD or aglycemia (Fig. 1e).

### Microglia after preconditioning with metabolic stress, exposed to a strong inflammatory stimulus

**Fig. 2:**
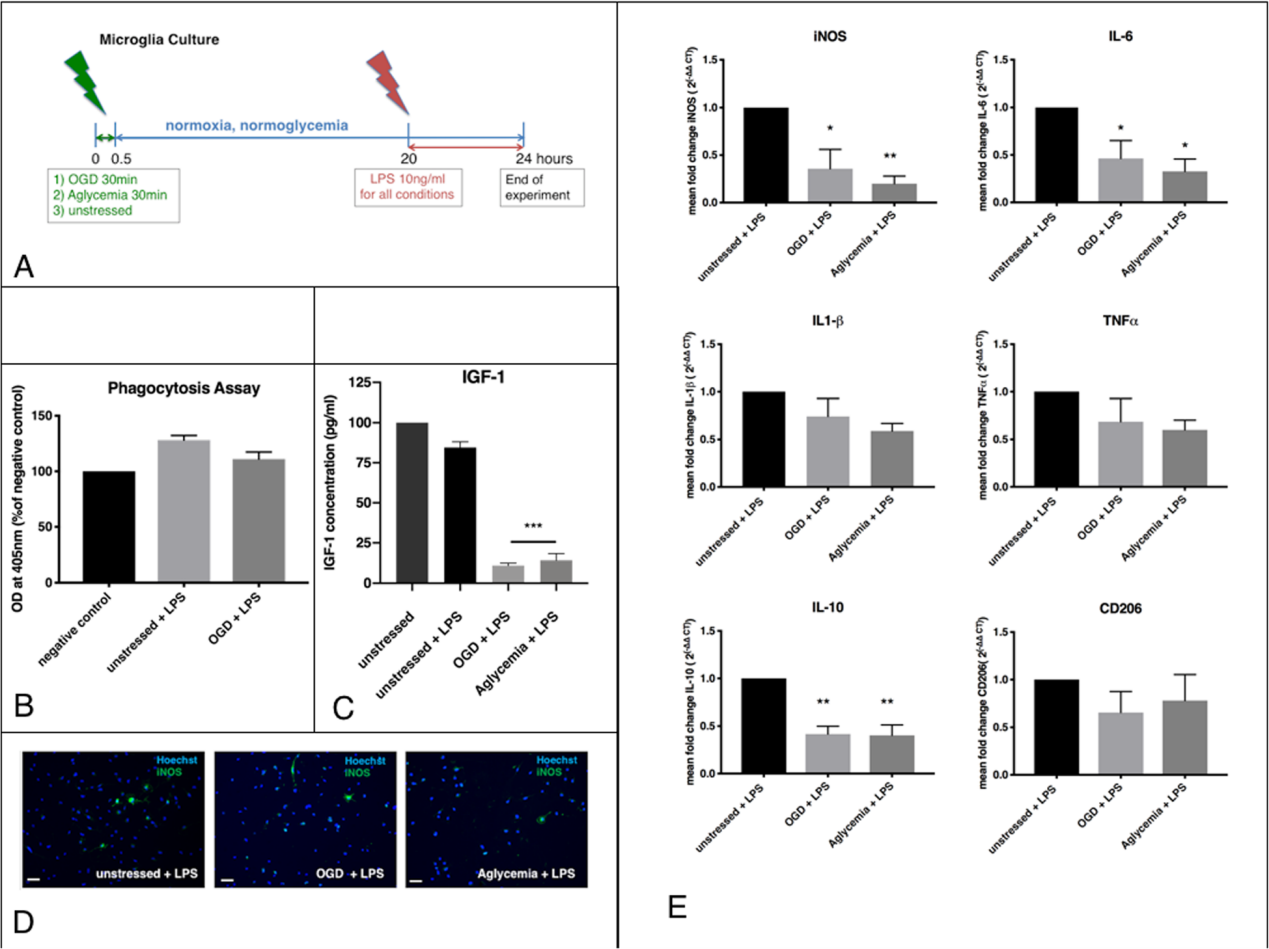
Microglia after preconditioning with metabolic stress, exposed to a strong inflammatory stimulus. **a** Experimental timeline. Microglia were exposed to metabolic stress by OGD or aglycemia for 30 min, and then allowed to recover in regular culture medium for 20 h. Subsequently, LPS as a robust inflammatory stimulus was applied at 10 ng/ml for 4 h. After this time, the cells were used for further experiments. Microglia treated with LPS served as controls. **b** Microglia were incubated with phagocytic beads (zymosan) for 2 h, and zymosan uptake was quantified photometrically. Untreated cells without zymosan incubation served as the negative control. There was no significant effect of OGD + LPS on the phagocytic activity of microglia compared to only LPS-treated cells (values displayed as means ± SEM of three independent experiments with *n* = 6/each condition). **c** An IGF1-ELISA was used to quantify IGF1 release in the cell supernatant photometrically. Both OGD + LPS and aglycemia+LPS treatment significantly reduced IGF1 release compared to only LPS-treated controls (values displayed as means ± SEM of three experiments with *n* = 12/each condition; the difference between treated groups: ***p < 0.0001 (one-way ANOVA/Tukey’s multiple comparison test). **d** Representative immunocytochemical images of iNOS+ cells (green), co-stained with a nuclear marker (Hoechst; blue). Staining for iNOS revealed less iNOS+ cells in microglia after OGD + LPS (middle panel) and in microglia after aglycemia+LPS (right panel) compared to only LPS-treated microglia (left panel) (scale bars = 50 μm). **e** Q-PCR revealed reduced expression of the markers iNOS, IL-6, and IL-10 after OGD + LPS, as well as after aglycemia+LPS, compared to LPS-only treated controls. Expression levels of TNFα, IL-1β, and CD206 did not differ between groups. Difference between OGD + LPS and aglycemia+LPS treated groups compared to LPS-treated control: *p < 0.05, ***p < 0.001 (values displayed as means ± SEM of one representative experiment of three independent experiments with at least *n* = 12/each condition; one-way ANOVA/Tukey’s multiple comparison test). Each Q-PCR sample was normalized to RPL13a as the reference gene, and mRNA levels were normalized to endogenous RPL13a expression

The microglia were exposed to 30 min of either OGD or aglycemia and then allowed to recover for 20 h at normoglycemic and normoxic conditions to examine the preconditioning effects of a metabolic stress before a strong inflammatory stimulus. The latter was induced by exposure to 10 ng/ml of LPS for 4 h (Fig. 2a). LPS-treated microglia without preconditioning served as controls. A phagocytosis assay assessed the microglia function after metabolic preconditioning and subsequent LPS stimulation. There was no significant change in phagocytotic activities between the preconditioned groups and non-preconditioned controls (Fig. 2b). However, metabolic preconditioning reduced IGF-1 levels significantly compared to non-preconditioned controls (Fig. 2c). Interestingly, total IGF-1 production was similar between untreated and LPS-treated controls. By contrast, there were less iNOS+ cells after preconditioning with either OGD or aglycemia, compared to non-preconditioned microglia (unstressed + LPS 5.6% iNOS + cells/total cell count +/− 2.4% SEM, OGD + LPS 1.8% iNOS + cells/total cell count +/− 0.5% SEM, aglycemia + LPS 2.0% iNOS + cells/total cell count +/− 0.6% SEM) (Fig. 2d). Quantification of pro- and anti-inflammatory markers by qPCR for mRNA levels of iNOS, TNFα, IL-6, IL1-beta, IL-10, and CD 206 revealed a significant downregulation of the pro-inflammatory markers iNOS and IL-6 after preconditioning. The anti-inflammatory marker IL-10 was also significantly reduced after preconditioning. The other pro-inflammatory markers TNFα and IL-1β were reduced only by trend after preconditioning, while CD206 levels remained unaffected (Fig. 2e).

### Effects of metabolically stressed neuronal/glial co-culture supernatant on microglia

**Fig. 3:**
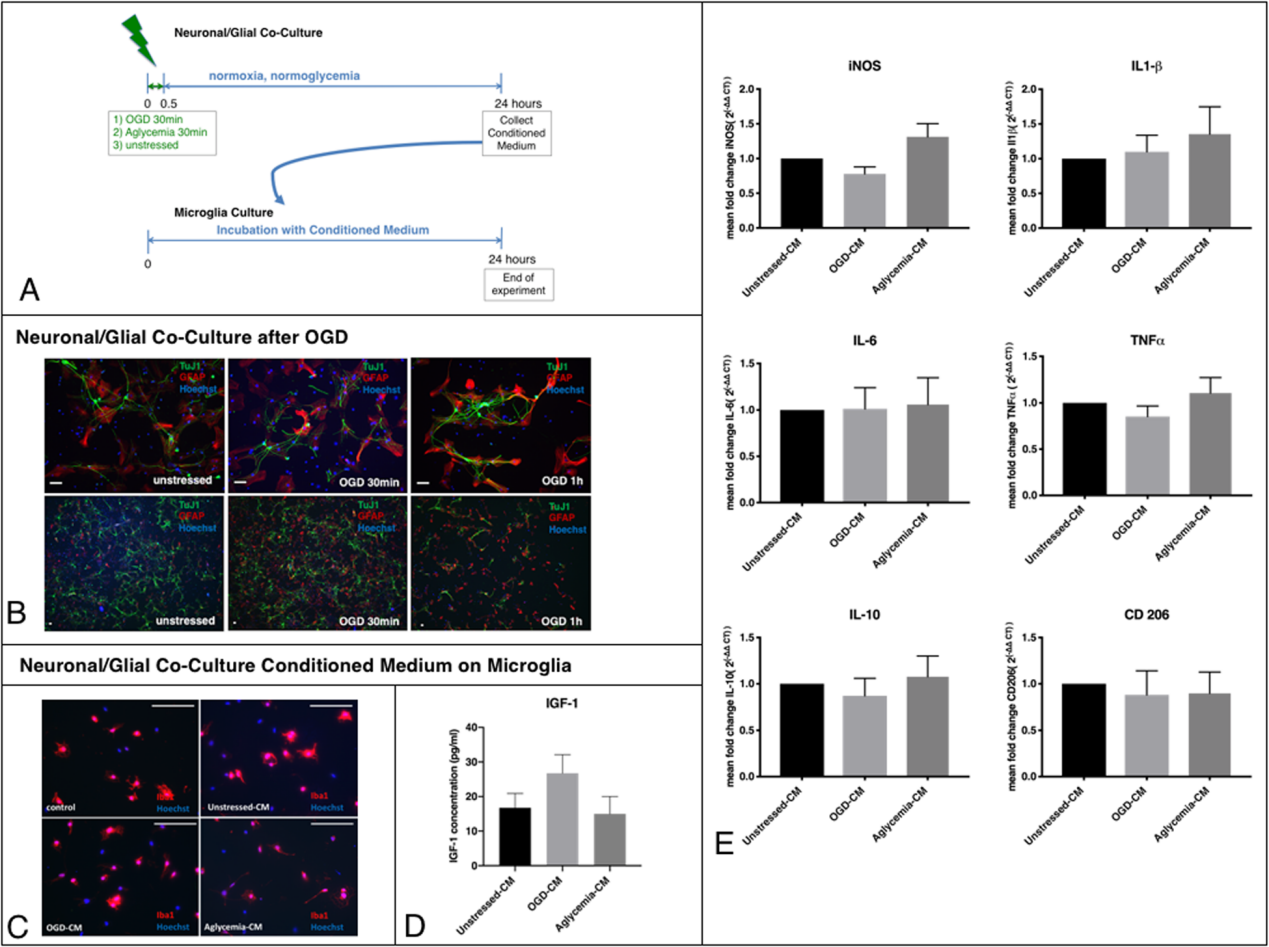
Effects of neuronal/glial co-cultures exposed to metabolic stress on microglia. **a** Experimental timeline. Neuronal/glial co-cultures differentiated from primary neural stem cells were exposed to metabolic stress, either as OGD or as aglycemia, for 30 min, before they were allowed to recover in regular culture medium for 24 h. The supernatant of preconditioned neuronal/glial co-cultures (conditioned medium, CM) was used for further experiments. Unstressed neuronal/glial co-cultures served as control. The CM of all conditions was incubated with microglia for 24 h. Then the microglia were used for further experiments. **b** Representative immunocytochemical images of neuronal/glial co-cultures after OGD. Tuj1+ neurons (green) and GFAP+ astrocytes (red) were co-stained with a nuclear marker (Hoechst; blue) in either unstressed microglia (left panel), microglia after 30 min of OGD (middle panel) or microglia after 1 h of OGD (right panel*;* scale bars = 50 μm), showing less viable cells after 1 h of OGD. **c** Representative immunocytochemical images of microglia incubated with the CM of pre-conditioned neuronal/glial co-cultures. Iba1+ microglia (red) were co-stained with a nuclear marker (Hoechst; blue) in either untreated microglia (upper left panel), microglia with unstressed-CM (upper right panel), or microglia with OGD-CM (lower left panel), or aglycemia-CM, respectively (lower right panel; scale bars = 50 μm), showing no changes in morphology under these different conditions. **d** An IGF1-ELISA was used to quantify IGF1 release in the microglia supernatant photometrically. There were no significant changes in IGF1 release between untreated control microglia and microglia incubated with either unstressed-CM and OGD-CM, or aglycemia-CM (values displayed as means ± SEM of three independent experiments with *n* = 12/each condition; one-way ANOVA). **e** Q-PCR revealed that neuronal/glial co-cultures exposed to metabolic stress did not affect the expression of either M1 or M2 markers on microglia: iNOS, TNFα, IL-6, IL1-β, IL-10, and CD 206 were unchanged compared to microglia incubated with non-preconditioned medium (values displayed as means ± SEM of three independent experiments with *n* = 12/ each condition, one-way ANOVA). Each Q-PCR sample was normalized to RPL13a as the reference gene, and mRNA levels were normalized to the endogenous RPL13a expression

To create a neuronal/glial co-culture as an “in vitro brain model,” primary NSC were differentiated for 14d (Fig. 3b). Co-cultures were exposed to metabolic stress by OGD or aglycemia, respectively (Fig. 3a). To determine the optimal duration of OGD on this co-culture system and under the attempt of mimicking cerebral ischemia, durations of 30 and 60 min of OGD were investigated. After 30 min of OGD, slight morphological changes were detected, while after 60 min of OGD, cell death occurred (Additional file 2: Figure S2A). Thirty minutes resp. 60 min of OGD did not alter the proportions of GFAP+ astrocytes and TuJ1+ neurons (Additional file 2: Figure S2B and S2C). Hence, either 30 min of OGD or aglycemia were chosen for all following experiments. After OGD or aglycemia, cells were allowed to recover for 20 h in their standard culture medium at normoxic and normoglycemic conditions. Then the medium was collected.

To investigate the crosstalk between neurons/glia and microglia, the microglia were exposed to conditioned medium (CM) from neuronal/glial co-cultures after OGD (OGD-CM), aglycemia alone (aglycemia-CM) or without exposure to metabolic stress (unstressed-CM). Afterwards, microglia were allowed to recover for 20 h at normoglycemic and normoxic conditions (Fig. 3a).

The number of viable microglia was unaffected by exposure to CM of neuronal/glial co-cultures, regardless of previous exposure to metabolic stress (Additional file 1: Figure S1B). Microglia morphology after 24 h of incubation in either medium or CM of the different conditions as assessed by Iba1 staining was unaffected (Fig. 3c). IGF-1 production as an anti-inflammatory marker was measured in the cell supernatant by ELISA and was unaltered in microglia exposed to CM from neuronal/glial co-cultures that had experienced metabolic stress (Fig. 3d). Quantification of pro- and anti-inflammatory markers was performed by qPCR for mRNA levels of iNOS, TNFα, IL-6, IL1-β, IL-10, and CD 206, and results were normalized to controls treated with unstressed-CM. Likewise, OGD-CM and aglycemia-CM did not affect expression levels of these inflammatory markers from microglia (Fig. 3e).

### Microglia preconditioned by crosstalk with metabolically stressed neuronal/glial co-cultures respond to a subsequent inflammatory stimulus

**Fig. 4:**
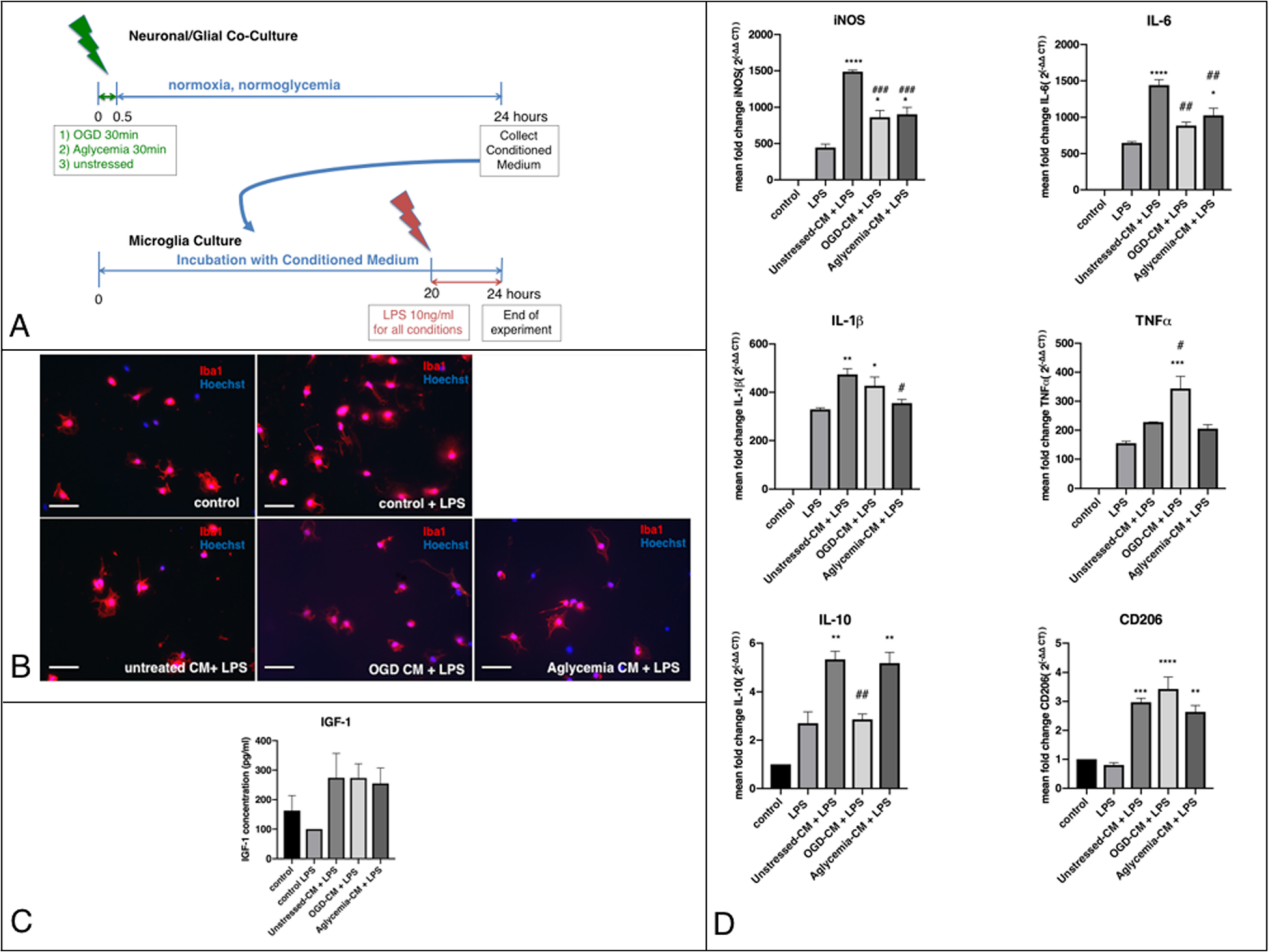
Microglia preconditioned by crosstalk with metabolically stressed neuronal/glial co-cultures respond to a subsequent inflammatory stimulus. **a** Experimental timeline. Neuronal/glial co-cultures were exposed to metabolic stress, namely OGD or aglycemia for 30 min and then allowed to recover in regular culture medium for 24 h. Next, their supernatant (conditioned medium, CM) was used for further experiments. Untreated neuronal/glial co-cultures served as controls. The CM of all conditions was incubated with microglia for 20 h, then—as a strong subsequent inflammatory stimulus—LPS was applied at 10 ng/ml for 4 h. After this time, cells were used for further experiments. Microglia treated with and without LPS incubated in the same cell culture medium as the CM served as controls. **b** Representative immunocytochemical images of microglia incubated with the CM of neuronal/glial co-cultures. Iba1+ microglia (red) were co-stained with a nuclear marker (Hoechst; blue) in either untreated microglia (upper left panel), untreated microglia with LPS (upper middle panel), microglia with unstressed-CM and LPS (lower right panel) or microglia with OGD-CM with LPS (lower middle panel), or aglycemia-CM with LPS (lower right panel; scale bars = 50 μm), showing no morphological changes in the different conditions. **c** An IGF1-ELISA was used to quantify IGF1-release in the cell supernatant photometrically. There were no significant changes in IGF1 release between controls with and without LPS and microglia incubated with unstressed-CM + LPS, OGD-CM + LPS, or aglycemia-CM+ LPS (values displayed as means ± SEM of three independent experiments with *n* = 12/each condition; one-way ANOVA). **d** Q-PCR revealed no significant differences for the phagocytosis marker CD68 between cells treated with LPS only and cells treated with unstressed-CM + LPS, OGD-CM + LPS, and aglycemia-CM + LPS (one-way ANOVA). Each Q-PCR sample was normalized to RPL13a as the reference gene, and mRNA levels were normalized to endogenous RPL13a expression. **e** Q-PCR revealed that incubation with unstressed-CM + LPS increased the expression of the markers iNOS, IL-6, IL-1β, and IL-10 compared to cells treated with LPS only. Treatment with OGD-CM and aglycemia-CM, however, decreased the expression of iNOS, IL-6, IL-1β (only aglycemia-CM), and IL-10 (only OGD-CM; values displayed as means ± SEM of one representative experiment of three independent experiments with at least *n* = 12/each condition; the difference between CM-treated groups compared to control with LPS: *p < 0.05, **p < 0.01, ***p < 0.001, ****p < 0.0001; the difference between OGD-CM and aglycemia-CM groups when compared to unstressed-CM group: ^#^*p* < 0.05, ^##^*p* < 0.01, ^###^*p* < 0.001; one-way ANOVA, Tukey’s multiple comparison test). Each Q-PCR sample was normalized to RPL13a as the reference gene, and mRNA levels were normalized to endogenous RPL13a expression

We next examined whether the secretome of metabolically stressed brain cells transferred to microglia by soluble factors in the medium would affect the response of microglia to a subsequent inflammatory stimulus. Microglia were exposed to CM of neuronal/glial co-cultures metabolically stressed either by OGD (OGD-CM) or aglycemia (aglycemia-CM). Microglia incubated with medium from unstressed neuronal/glial co-cultures (unstressed-CM) served as controls. Afterwards, microglia were stimulated with 10 ng/ml of LPS for 4 h (Fig. 4a). Microglia morphology was assessed by Iba1-staining. There were no morphological changes between the different conditions (Fig. 4b). There was a trend of increase in IGF-1 levels after treatment with CM + LPS as measured by ELISA (Fig. 4c). Quantification of pro- and anti-inflammatory markers by qPCR for mRNA levels of iNOS, TNFα, IL-6, IL1-β, IL-10, and CD 206 revealed significant upregulation of the pro-inflammatory markers iNOS, IL-6, and IL1-β after unstressed CM + LPS, compared to LPS alone. Results were normalized to microglia without CM and LPS treatment. Treatment with OGD-CM and aglycemia-CM led to a significant reduction of iNOS, IL-6, and IL1-β (the latter after aglycemia-CM only). For TNFα, only OGD-CM + LPS led to a significant upregulation compared to LPS treatment alone. IL-10 was regulated with similar dynamics as the proinflammatory cytokines, with an upregulation after unstressed-CM + LPS and a downregulation after OGD-CM + LPS. All CM conditions led to an upregulation of CD 206 (Fig. 4e). Additionally, the phagocytosis marker CD68 was measured. There were no significant differences in CD68 mRNA levels between microglia treated with LPS alone and microglia treated with the different CM conditions + LPS ((Fig. 4d).

## Discussion

Under the hypothesis that a crosstalk between microglia and other cells of the brain is essential to microglia activation and polarization, and that microglia “memorize” this crosstalk, resulting in divergent responses to subsequent stimuli, we used an in vitro model of cerebral ischemia, i.e., oxygen-glucose deprivation (OGD), to investigate direct and indirect effects of this metabolic stress on microglia and their ability to respond to a robust inflammatory stimulus.

Interestingly, metabolic stress by OGD or aglycemia alone did not affect primary microglia function or their expression of inflammatory markers. In the literature, pro-inflammatory effects of OGD on primary microglia in culture have been described [14, 15], albeit sometimes in a shallow range [16] and with a heterogeneous response of different pro-inflammatory markers [17]. In line with our results, another study also did not observe significant changes in inflammatory markers in primary microglia after OGD [18]. Taken together, literature data on microglia OGD is contradictory, which may be attributed to the heterogeneity of primary microglia and methodical differences in the OGD setting [14–18] as well as species differences [19]. Studies on aglycemia in microglia are scarce, and thus far only two studies focused on this topic, finding an increased pro-inflammatory response of microglia after aglycemia [20, 21]. Given the resistance of microglia to metabolic stress by OGD or aglycemia, we further tested if ischemic resp. aglycemic preconditioning altered the response to a subsequent robust inflammatory stimulus. In microglia, such a strong inflammatory stimulus can be achieved by LPS stimulation [2]. Results show that both OGD and aglycemia alter microglia to release less pro-inflammatory cytokines in response to LPS as a “second-hit” stimulus. Interestingly, the release of the anti-inflammatory cytokine IL-10 was also reduced after preconditioning, while CD206 levels remained unaffected.

Preconditioning describes the induction of tolerance by a first mild stimulus against a later stronger stimulus. The concept of ischemic preconditioning is well accepted for neurons, astrocytes, and microglia [22–28]. The idea of beneficial preconditioning has been translated to the in vivo situation of experimental stroke (for a review, see [29]). Even in the situation of human stroke, preconditioning phenomena may occur [30, 31]. Non-ischemic preconditioning with LPS also reduces sensitivity to a subsequent ischemic event [32, 33]. Furthermore, non-ischemic preconditioning with pharmacological agents such as isoflurane, hypo- and hyperthermia, or caloric restriction has been described [34]. Effects of preconditioning are acutely mediated via DAMP and TLR4. Moreover, transient downregulation of the innate immune system has been reported [32, 35]. In microglia, IFN signaling has been attributed to ischemic tolerance [26]. However, to the best of our knowledge, this is the first report describing that ischemic preconditioning also vice versa reduces the sensitivity to a LPS stimulus, in line with a “second hit” injury. Additionally, transient aglycemia is capable of inducing preconditioning.

Unlike conditions in vivo, culturing microglia in the absence of other cells of the central nervous system (CNS) is far from the physiological situation [36]. For this reason, we here investigated how crosstalk from other brain cells—whether unstressed or after the experience of metabolic stress—affected microglia. Interestingly, we found that the preconditioning effects of metabolic stress were transferred from a neuronal/glial co-culture to microglia by soluble factors in the conditioned medium. However, this crosstalk alone did not activate or change microglia (cf. Fig. 3) but increased their responsiveness to a subsequent inflammatory LPS stimulus (cf. Fig. 4). Several reports described a proinflammatory activation of microglia by treatment with CM from neurons that underwent OGD [37–40], CM from endothelial cells after OGD [18], or microglia in a neuronal-microglia co-culture [41]. In contrast, the CM of OGD-pretreated astrocytes induces a mostly anti-inflammatory phenotype in microglia [18, 42]; however, a recent study also described the induction a proinflammatory phenotype [43]. In our mixed neuronal/glial co-culture, effects of OGD on neurons could have been cleared by astrocytes [28, 44] or anti-oxidative mechanisms could have been induced reciprocally [45]. Thus, we propose that our mixed neuronal/glial co-culture is much closer to an “in vitro model of the brain” than an isolated neuron culture.

The “M2” markers CD 206, IL-10, and IGF-1 showed a heterogenous response throughout the experiments. While IL-10 dynamics were comparable to the M1 markers, IGF-1 and CD 206 showed specific changes. The latter is in line with previous findings from our group showing diverging dynamics of M2-markers in vivo after experimental stroke [46]. IGF-1 was reduced after OGD and aglycemia without any influence of additional LPS stimulation. IGF-1 is an important neurotrophic growth factor mainly produced by microglia [47]. Chronic neuroinflammation leads to a suppression of IGF-1 [48, 49]. Therefore, reduced levels of IGF-1 due to metabolic stress in the form of OGD or aglycemia may lead to the reduction of trophic support. However, acute inflammation in the form of a LPS stimulus did not affect IGF-1 levels. This points to the fact that M2 markers represent a large scale of different effectors and cannot be compared directly [3].

Interestingly, the two models of metabolic stress chosen here, OGD and aglycemia, were quite comparable in their effects, which may suggest that their effects result from the metabolic stress rather than the hypoxia. In contrast to the study of Choi et al., we here observed similar responses of microglia to OGD and aglycemia [20]. Encompassing their study, we examined several pro-inflammatory and anti-inflammatory markers on microglia. Unlike the study of Churchward et al., we did not study a simultaneous LPS stimulus during aglycemia, but studied the influence of LPS 24 h after aglycemia [21]. While Churchward et al. found a boost of the proinflammatory response during simultaneous aglycemia/LPS stimulation, we instead observed a pre-conditioning effect of aglycemia on a subsequent—but independent—LPS stimulus.

Taken together, metabolic stress in the form of OGD or aglycemia elicits a specific microglia signature characterized by a decreased inflammatory response to subsequent inflammation. This effect can be evoked by metabolic stress not only directly applied to microglia, but also indirectly by mediation through other cells of the brain via soluble factors.

## Conclusions

Data suggest a specific microglia signature in response to ischemic and aglycemic preconditioning and a subsequent inflammatory stimulus.

## Supplementary information

**Additional file 1: Figure S1.** A. Aglycemia for 30 min did not lead to cell death as measured by the number of propidium iodide positive microglia, while OGD at a longer duration of 45 min led to significantly increased cell death compared to unstressed microglia. **p < 0.01; one-way ANOVA, Tukey’s Multiple Comparison Test (values displayed as means ± SEM of three experiments). B. The number of nonviable microglia, as measured by the number of propidium iodide positive microglia, was unaffected by exposure to CM of neuronal/glial co-cultures, regardless of previous exposure to OGD (values displayed as means ± SEM of two experiments; one-way ANOVA).

**Additional file 2: Figure S2.** A. After 30 min of OGD cell numbers were not significantly reduced, while after 60 min of OGD, cell death occurred resulting in reduced cell numbers compared to control: *p < 0.05 (values displayed as means ± SEM of one representative experiment of two experiments; one-way ANOVA, Tukey’s Multiple Comparison Test). B. The percentage of GFAP+ astrocytes of the total cell count was not affected by OGD of 30 and 60 min (values displayed as means ± SEM of one representative experiment of two experiments; one-way ANOVA). C. The percentage of Tuj1+ neurons of the total cell count was not affected by OGD of 30 and 60 min (values displayed as means ± SEM of one representative experiment of two experiments; one-way ANOVA).

## Data Availability

The datasets generated and analyzed during the current study are available from the corresponding author on reasonable request.

## Abbreviations

ANOVA: One-way analysis of variance
CM: Conditioned medium
CNS: Central nervous system
iNOS: Inducible nitrite oxygen synthesis
LPS: Lipopolysaccharides
OD: Optical density
OGD: Oxygen-glucose deprivation
RT-qPCR: Real-time quantitative PCR
