# Supplementary figures and images for: Crosstalk between stressed brain cells: direct and indirect effects of ischemia and aglycemia on microglia
Source: J Neuroinflammation. 2020 Jan 24;17:33. doi: 10.1186/s12974-020-1697-8 (PMC6982395; doi:10.1186/s12974-020-1697-8)

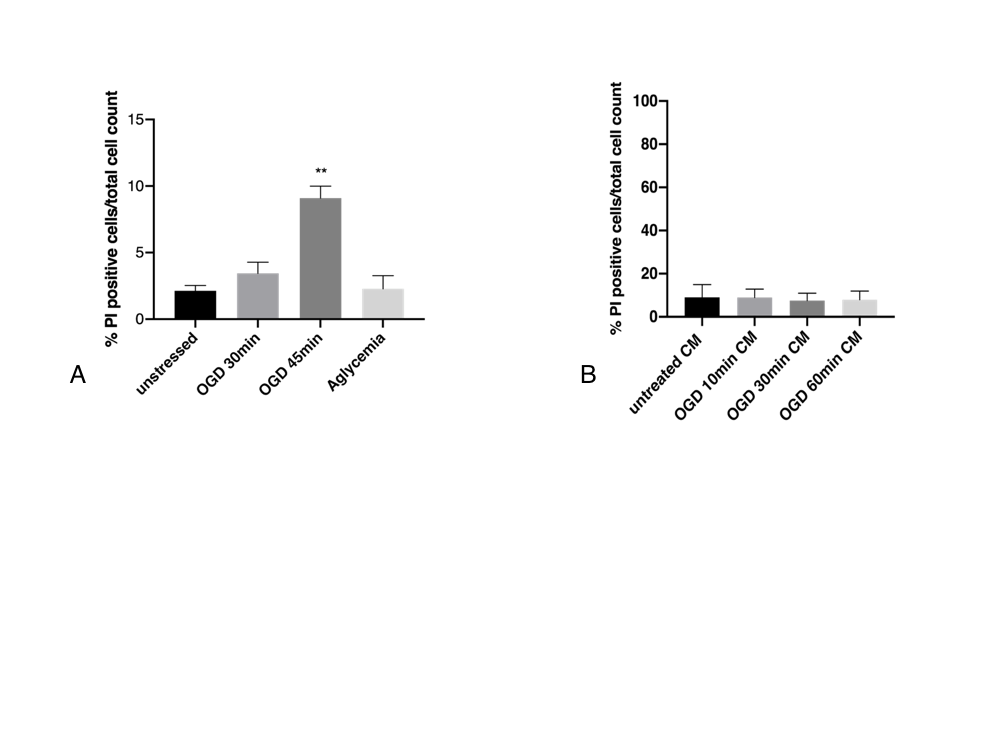

Supplement: Supplementary file 1 — Additional file 1: Figure S1. A. Aglycemia for 30 min did not lead to cell death as measured by the number of propidium iodide positive microglia, while OGD at a longer duration of 45 min led to significantly increased cell death compared to unstressed microglia. **p < 0.01; one-way ANOVA, Tukey’s Multiple Comparison Test (values displayed as means ± SEM of three experiments). B. The number of nonviable microglia, as measured by the number of propidium iodide positive microglia, was unaffected by exposure to CM of neuronal/glial co-cultures, regardless of previous exposure to OGD (values displayed as means ± SEM of two experiments; one-way ANOVA). [file 12974_2020_1697_MOESM1_ESM.tiff]

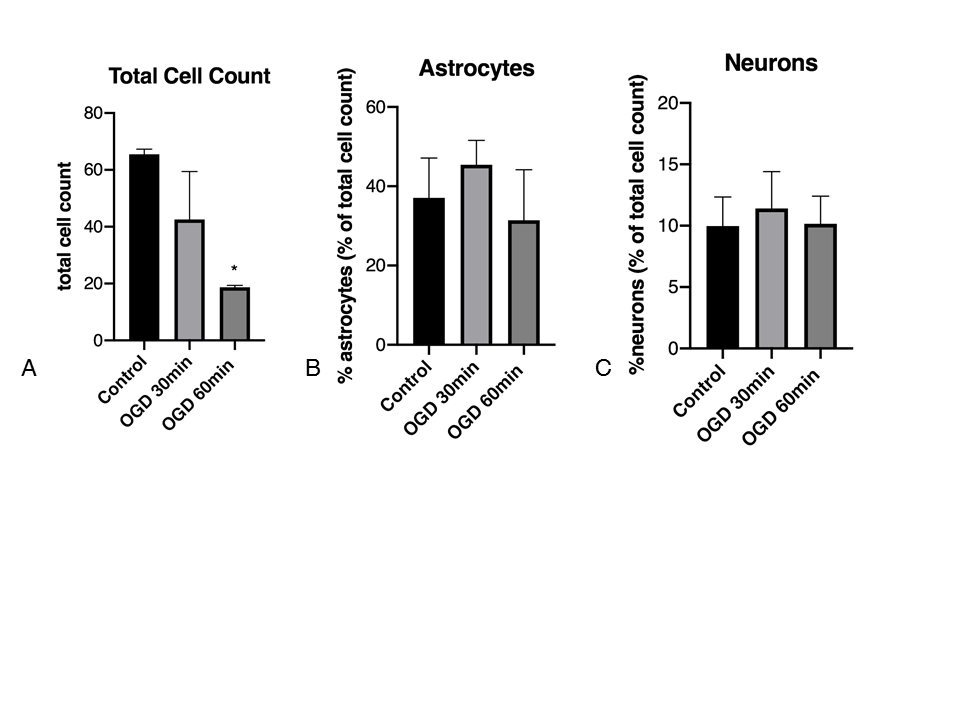

Supplement: Supplementary file 2 — Additional file 2: Figure S2. A. After 30 min of OGD cell numbers were not significantly reduced, while after 60 min of OGD, cell death occurred resulting in reduced cell numbers compared to control: *p < 0.05 (values displayed as means ± SEM of one representative experiment of two experiments; one-way ANOVA, Tukey’s Multiple Comparison Test). B. The percentage of GFAP+ astrocytes of the total cell count was not affected by OGD of 30 and 60 min (values displayed as means ± SEM of one representative experiment of two experiments; one-way ANOVA). C. The percentage of Tuj1+ neurons of the total cell count was not affected by OGD of 30 and 60 min (values displayed as means ± SEM of one representative experiment of two experiments; one-way ANOVA). [file 12974_2020_1697_MOESM2_ESM.png]
